# The application of the radiomic-clinical model based on SHAP-XGBoost method for differentiating pulmonary tuberculosis from *Streptococcus pneumoniae* pneumonia in children

**DOI:** 10.3389/fmed.2026.1838680

**Published:** 2026-06-04

**Authors:** Min Lei, Chunjiao Han, Yulian Fang, Zhiheng Xing, Chunquan Cai

**Affiliations:** 1 Department of Pediatric Research Institute, Children’s Hospital, Tianjin University/Tianjin Children’s Hospital, Tianjin, China; 2 Tianjin Key Laboratory of Birth Defects for Prevention and Treatment, Children’s Hospital, Tianjin University/Tianjin Children’s Hospital, Tianjin, China; 3 Department of Radiology, Haihe Hospital, Tianjin University, Tianjin, China; 4 Tianjin Institute of Respiratory Diseases, Tianjin, China

**Keywords:** machine learning, predictive model, pulmonary tuberculosis, radiomics, *Streptococcus pneumoniae* pneumonia

## Abstract

**Purpose:**

Diagnosing pulmonary tuberculosis (PTB) in children remains challenging due to its often non-specific clinical presentation. Early detection enables timely treatment initiation. This study aimed to develop and validate a predictive model to distinguish PTB from *Streptococcus pneumoniae* pneumonia (SPP) in children.

**Methods:**

Lung CT images were obtained from 52 children with PTB and 80 with SPP, from which 1,023 radiomic features were extracted. The data were randomly split into training and test sets at a 7:3 ratio. Variance threshold, univariate analysis, and LASSO regression were applied sequentially to select the key radiomic features. Subsequently, an XGBoost model integrating clinical factors and radiomic features was constructed. The SHAP method was used to interpret the model and its decision process.

**Results:**

After feature selection, six radiomic features and three clinical features were retained for model construction. The combined radiomic-clinical model outperformed both the clinical-only and radiomic-only models. Its AUC was 0.981 in the training cohort and 0.897 in the test cohort. The clinical model achieved AUCs of 0.955 and 0.784, and the radiomic model achieved 0.868 and 0.865, respectively. SHAP summary and waterfall plots illustrated the contribution of each feature to the predictions.

**Conclusion:**

The SHAP-XGBoost radiomic-clinical model accurately distinguished PTB from SPP in children. It may offer a non-invasive and efficient tool to support clinical decision-making.

## 1 Introduction

The WHO Global Tuberculosis Report 2023 indicates that, in 2022, newly diagnosed pediatric tuberculosis (TB) accounted for approximately 12% of all global TB cases. Notably, children represented about 16% of total TB-related deaths. Because the signs and symptoms of pulmonary TB (PTB) in children are often non-specific and can resemble those of community-acquired pneumonia (CAP), delayed or incorrect diagnoses are common in this population (1, 2).

In developing countries, CAP ranks among the leading causes of pediatric hospitalization and mortality (3, 4). Although microbiological testing plays a key role in identifying the etiology and guiding treatment for CAP, determining the specific microbial cause remains difficult in many clinical settings (5, 6). *Streptococcus pneumoniae* is the predominant bacterial pathogen responsible for CAP in children (7), and the morbidity and mortality from lower respiratory tract infections in this age group are largely attributable to *S. pneumoniae* pneumonia (SPP) (8). Early control of pulmonary inflammation has been proposed as the most promising approach to reduce mortality (9).

Radiologically, PTB and SPP share several overlapping features, including consolidation, nodular opacities, and ground-glass attenuation (10). Although both diseases are caused by bacteria, their treatment regimens differ markedly. Currently available pathogen detection methods have limitations, including long turnaround times, false positives, and false negatives, which hamper rapid etiological diagnosis (11). Therefore, timely differentiation between PTB and SPP is essential for the appropriate use of antibiotics and anti-tuberculosis drugs. The objective of this study was to develop and validate a predictive model capable of effectively distinguishing PTB from SPP in routine clinical practice.

## 2 Materials and methods

### 2.1 Patients and definitions

Lung CT images were retrospectively collected from patients with tuberculosis and pneumonia in our hospital between January 2018 and December 2023. The inclusion criteria were as follows: (a) The condition met the diagnostic criteria for PTB and SPP; (b) Children had not received antitubercular therapy (ATT) prior to enrollment. (c) Children did not receive antibiotic treatment before admission. The exclusion criteria were as follows: (a) Age older than 18 years; (b) Presence of underlying chronic diseases; (c) Incomplete clinical information or imaging data. The diagnostic criteria for PTB were as follows (12, 13): (1) Having symptoms and signs related to pulmonary tuberculosis. (2) Imaging is consistent with active pulmonary tuberculosis. (3) Tuberculin skin test (TST) and/or interferon-gamma release assays (IGRAs) positive. (4) Phlegm, induced sputum, bronchoalveolar lavage fluid, gastric juice, pleural effusion, tissue samples, etc. are positive for acid fast staining; MTB culture, molecular biology testing for MTB nucleic acid, or pathological results are positive. The diagnostic criteria for SPP were as follows: (1) Diagnostic criteria for bronchopneumonia (14): presence of respiratory symptoms such as fever, cough, and shortness of breath, auscultation of the lungs with audible mid to fine moist rales, and/or imaging showing inflammatory changes in the lungs. (2) The pathogen test of SP is positive (pleural effusion or blood culture). Written informed consents were obtained from the parents for publication of this report.

### 2.2 CT examination

All lung CT scans were performed using a Philips iCT scanner. The scanning included imaging from the entrance of the chest to the bottom of the lungs with deep inspiration breath-hold. The scanning parameters were as follows: tube voltage, 120 kV; automatic tube current modulation; detector collimation, 128 × 0.625 mm; rotation time, 330 ms; and pitch, 0.758. The image reconstruction parameters were as follows: slice thickness, 2 mm; increment, 1 mm; field of view, 20 cm; and matrix, 512 × 512. Then, the reconstructed images were transferred to 3D Slicer^1^ for radiomics analysis. Phylogenetic and amino acid mutation analysis.

### 2.3 Pulmonary consolidation segmentation

**Figure 1:**
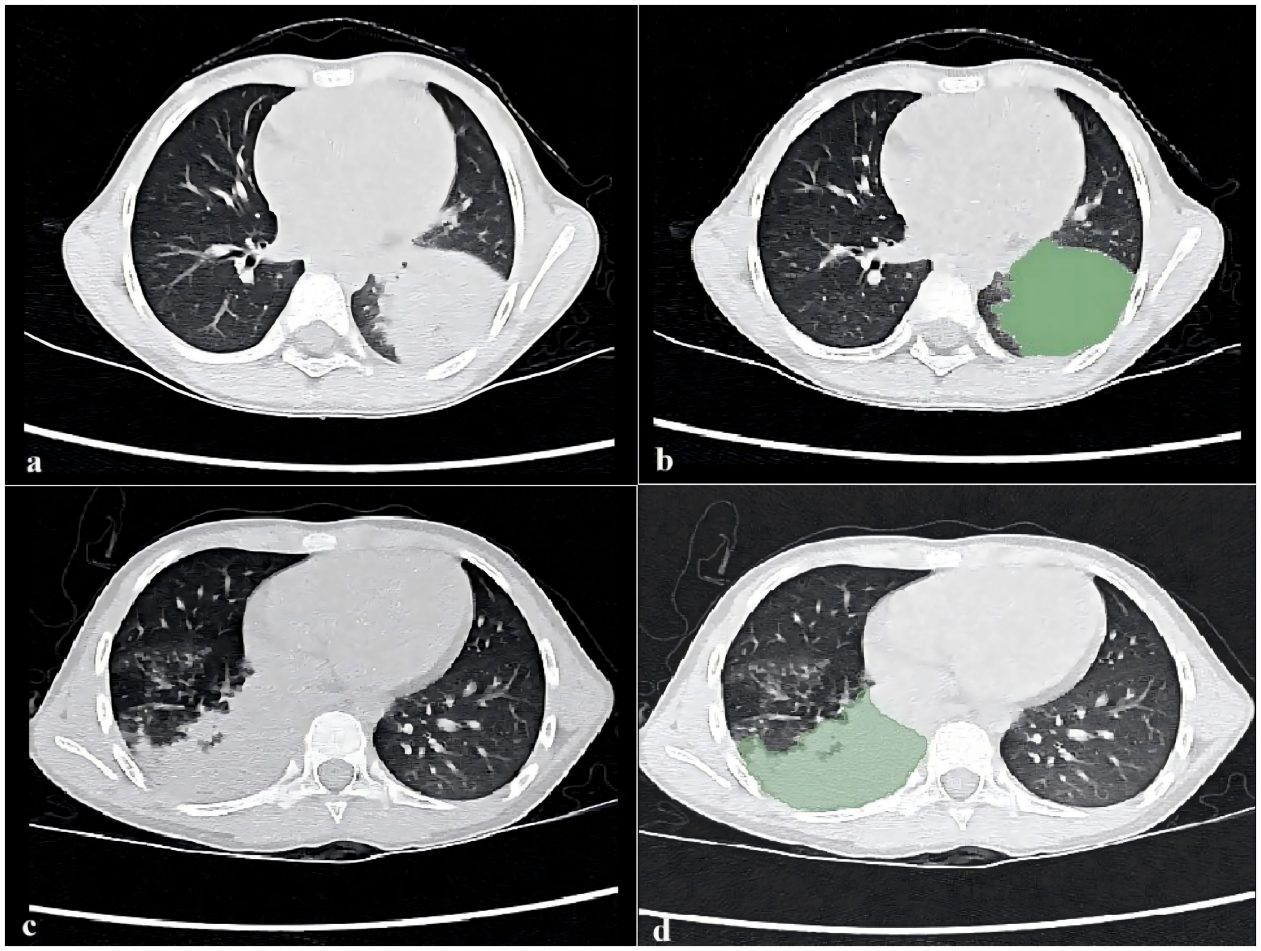
**(a,b)** Lung window from a 5-year female child with SPP. **(c,d)** Lung window from a 6-year-old female child with PTB. The area of pulmonary consolidation (green mark) was manually delineated in each patient.

The area of pulmonary consolidation was manually segmented by an experienced radiologist by 3D Slicer software and confirmed by another experienced radiologist (Figure 1). Both radiologists were blinded to the diagnosis of children. We calculated the intraclass correlation coefficient (ICC) for the radiomic features extracted from the first and second delineations. Features with ICC > 0.80 were retained.

### 2.4 Radiomics feature extraction

The radiomics features were extracted from each ROI by 3D Slicer software. For each filter, six classes of radiomic features were extracted: GLDM, GLCM, first-order, GLRM, GLSZM, and NGTDM.

### 2.5 Model construction

The training and validation sets were randomly sampled in a 7:3 ratio. All feature selection steps were performed exclusively on the training set. From the 1,023 extracted radiomic features, variance threshold (0.8), univariate analysis (*p* < 0.05), and LASSO regression were sequentially applied to identify key radiomic features. For clinical features, univariate analysis (*p* < 0.05) was used to select significant variables.

**Figure 2:**
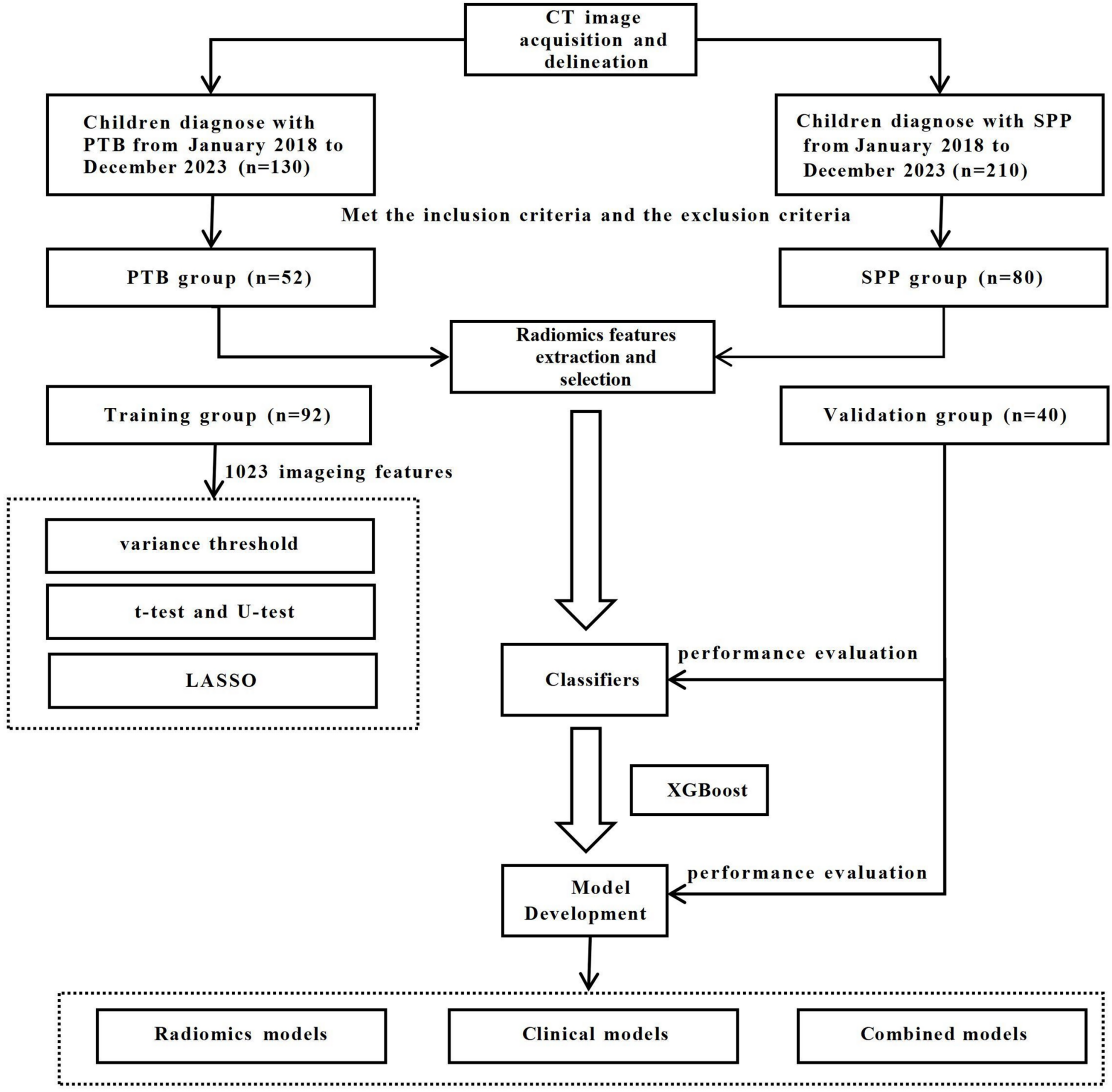
Flowchart of the whole study.

We further established three XGBoost classifiers, including a radiomic model using the filtered radiomic features alone, a clinical model constructed with only significant clinical characteristics, and a joint model combining radiomic and clinical features. Hyperparameters were tuned via five-fold cross-validation on the training set. The overall process is illustrated in Figure 2.

### 2.6 Statistics analysis

Statistical analyses were conducted using Python 3.6.8, R 4.3.2, and SPSS 26.0. Normally distributed data were expressed as mean ± standard deviation or mean ± 95% confidence interval (CI). The independent sample *t* test was used for comparison. Non-normally distributed data were described as median (P25, P75), and comparisons were made using the Mann–Whitney *U* test. The chi-square test was used for categorical data. A *p* value < 0.05 was considered statistically significant.

## 3 Results

### 3.1 Clinical characteristics

The modeling set comprised 92 children: 36 with PTB and 56 with SPP. Based on univariate analysis, three variables—maximum temperature, illness duration, and cough—reached statistical significance (*p* < 0.05). Table 1 summarizes the clinical characteristics of both groups.

**Table 1:** Clinical characteristics of children with PTB and SPP.

Characteristic	PTB group	SPP group	Total	*p*-value
	(*n* = 36)	(*n* = 56)	(*n* = 92)	
Age (years)	8 (4, 12)	7 (5, 9)	7.5 (4.3, 9.8)	0.280
Fever duration	1.5 (0, 8.5)	4 (2, 5)	4 (1, 6)	0.314
Tmax (°C)	38.5 (36, 39.2)	39.3 (38.6, 39.7)	39 (38.3, 39.6)	0.000
Illness duration	10 (5.25, 30)	5.5 (4, 10)	7 (4.25, 13.25)	0.004
Gender				1.000
Male	18	28	46	
Female	18	28	46	
Cough	28	56	82	0.001
Wheeze	2	6	8	0.633
Chest pain	3	2	5	0.609
Abdominal pain	3	5	8	1.000
Weight loss	7	2	9	0.864

### 3.2 Radiomics feature selection

**Figure 3:**
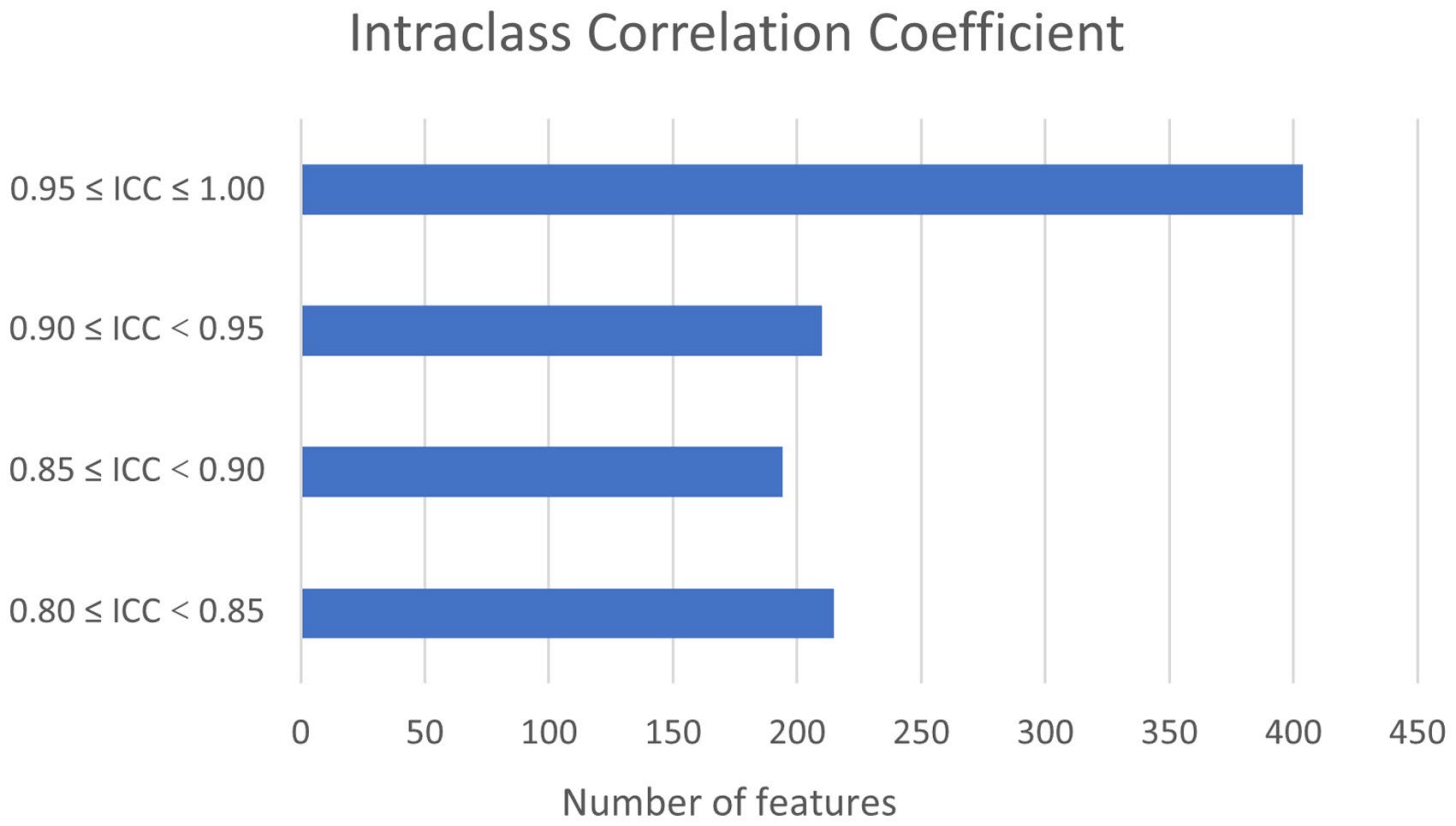
Distribution of intraclass correlation coefficients (ICC) for the 1,023 radiomic features.

**Figure 4:**
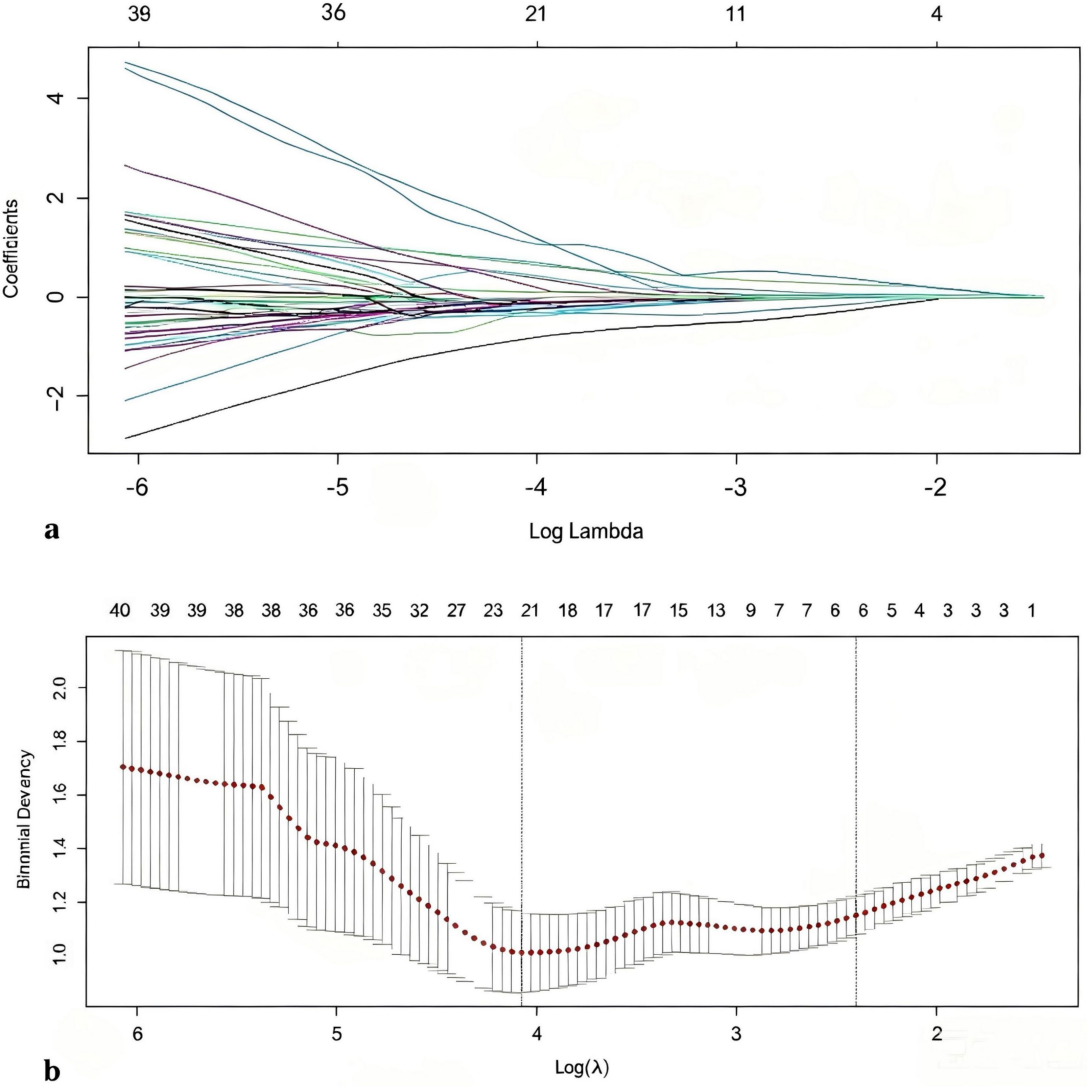
**(a)** Lasso regression coefficient path diagram. The *x*-axis represents the value of regularization parameters. The *y*-axis represents the size of model parameters. Each curve or path corresponds to a feature in the model. **(b)** Lasso regression cross-validation curve. The *x*-axis represents the values of regularization parameters. The *y*-axis typically represents the performance metrics of the model. The vertical line on the left represents lambda min and the right represents lambda 1se.

A total of 1,023 radiomic features were extracted from the CT images of pulmonary consolidation with ICC > 0.80. The ICC distribution of the 1,023 radiomic features is shown in Figure 3. The variance threshold method selected 542 features. Further screening using independent-sample t-tests and Mann–Whitney *U* tests yielded 210 features. Finally, LASSO regression identified six key features for differentiating PTB from SPP (Figure 4). All six features showed significant differences between the two groups in univariate analysis (*p* < 0.05). Detailed information for these features is presented in Table 2.

**Table 2:** Six radiomic features selected by LASSO.

Characteristic	Radiomic feature	Radiomic class	Filter	Coefficient
X19	Gray level non-uniformity	glszm	log-sigma-4-0-mm-3D	0.02174
X48	Cluster shade	glcm	wavelet-LHL	−0.07835
X76	Cluster prominence	glcm	wavelet-LHH	0.25913
X159	Mean	firstorder	wavelet-HHL	−0.20363
X187	Contrast	glcm	original	0.32748
X188	Difference variance	glcm	original	0.06087

### 3.3 Machine learning methods

**Figure 5:**
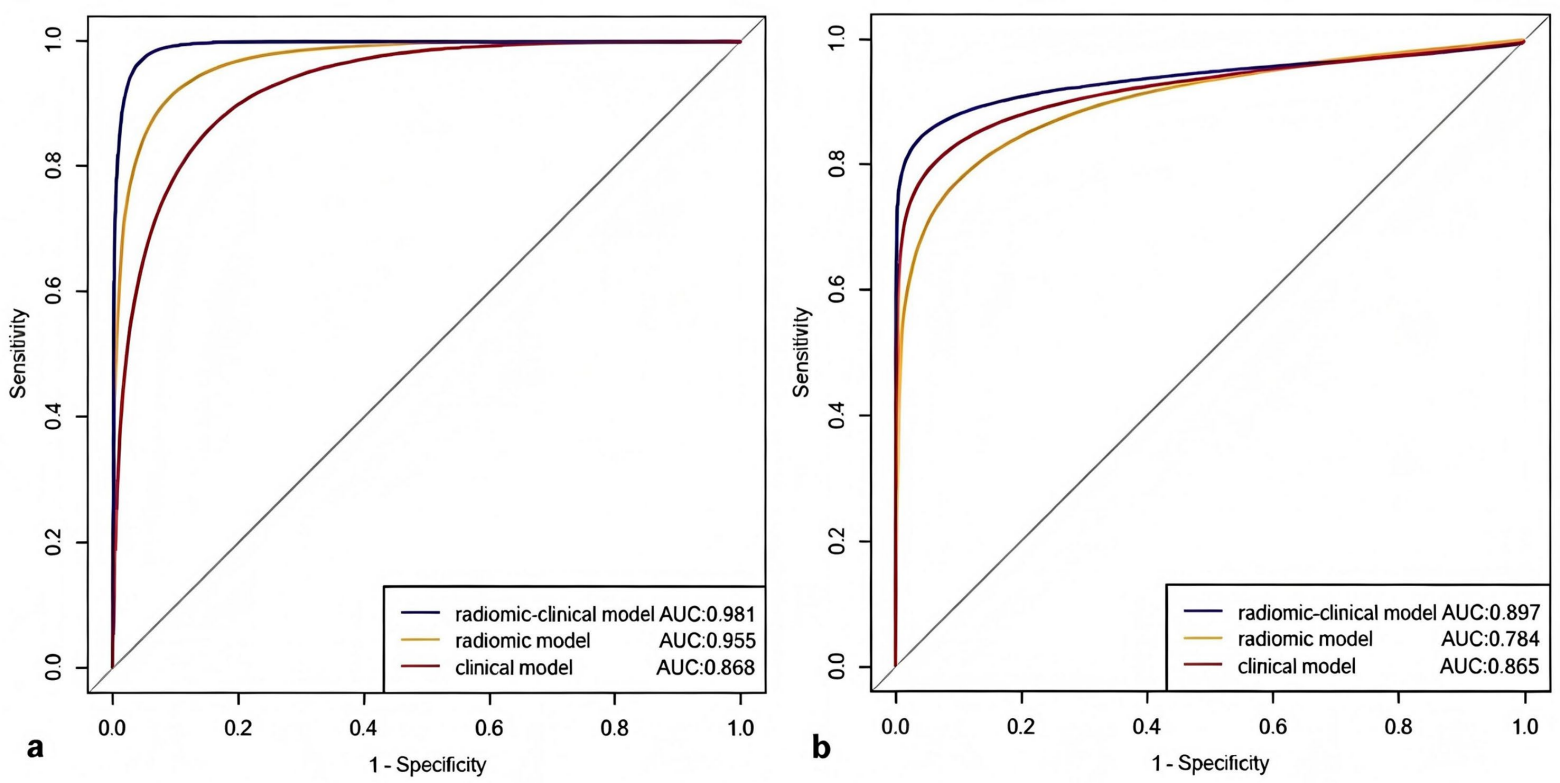
Comparison of receiver operating characteristic curves for assessing the radiomic model, clinical model, and radiomic-clinical model in the training cohort **(a)** and validation cohort **(b)**.

The ROC curves for the three models are presented in Figure 5. The radiomic-clinical model outperformed the other two in both the training and test sets. In the training cohort, its AUC reached 0.981, and in the test cohort, 0.897. Accuracy was 0.946 for training and 0.825 for testing. Table 3 provides a detailed breakdown.

**Table 3:** Performances of model to differentiate PTB from SPP in the training cohort and validation cohort.

Model	Cohort	AUC (95%CI)	Sensivity	Specifity	Precision	Accuracy	F1
Radiomic-clinical model	Training cohort	0.878–0.982	0.946	0.946	0.921	0.946	0.933
	Testing cohort	0.672–0.927	0.600	0.960	0.900	0.825	0.720
Radiomic model	Training cohort	0.809–0.947	0.865	0.909	0.865	0.891	0.865
	Testing cohort	0.616–0.892	0.533	0.920	0.800	0.775	0.640
Clinical model	Training cohort	0.783–0.931	0.706	0.966	0.923	0.870	0.800
	Testing cohort	0.644–0.910	0.611	0.955	0.917	0.800	0.733

### 3.4 Explanation and visualization of XGBoost model with the SHAP method

**Figure 6:**
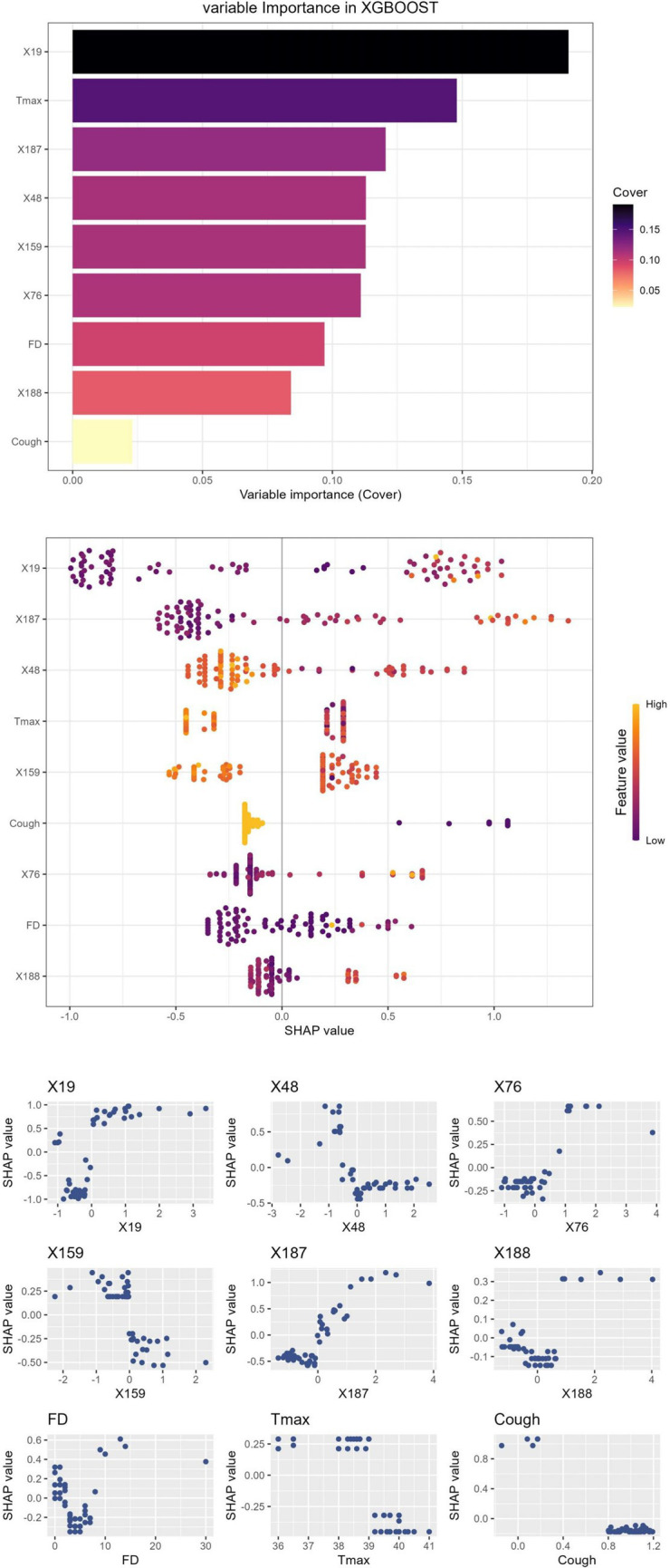
**(a)** SHAP column chart. The importance ranking of each variable according to the mean (|SHAP value|). **(b)** SHAP summary plot. Plots represent the correlation and contribution between features and model predictive ability. **(c)** SHAP scatter plot. Clearly demonstrate the direction, magnitude, and variation of the impact of features on model predictions.

We used SHAP to visualize the radiomic-clinical model built with XGBoost. Figure 6a shows the importance ranking of each variable in the model. The feature log-sigma-4-0-mm-3D came out as the most important for distinguishing PTB from SPP. In Figure 6b, the SHAP method was applied to show the positive and negative relationships of each variable. For log-sigma = 4–0 = mm-3D, the color indicated a positive impact on the model’s output, while higher values of Tmax and cough had a negative impact. Figure 6c presents the SHAP values for each variable based on prediction probability. This plot reveals noticeable changes in the feature values corresponding to the model’s predictions.

## 4 Discussion

In this study, we built a machine learning model that combined radiomics signatures with clinical features using XGBoost. The goal was to tell PTB apart from SPP in children. The model performed well in both training and validation sets, achieving AUCs of 0.981 and 0.897, respectively. This outperformed the models using clinical features alone or radiomic features alone. This model may assist clinicians in making more accurate diagnoses.

Some previous studies have looked at clinical prediction models for pediatric PTB. Gunasekera et al. (15) built a regression model to assess the contribution of baseline clinical evaluation. Their clinical model, based solely on initial clinical evidence, achieved an AUC of 0.75; adding chest radiographic findings and Xpert MTB/RIF results increased the AUC to 0.87. Smith et al. (16) applied machine learning to predict culture-confirmed TB in children under 5 years. Their models performed reasonably well for samples from invasive procedures (AUC 0.84–0.90) and non-invasive ones (0.83–0.89). Brooks et al. (17) developed two clinical prediction tools—one using classification and regression tree (AUC 0.949) and another using logistic regression (AUC 0.985)—to identify children who might benefit from rapid initiation of TB treatment.

Most radiomics studies on pulmonary TB have focused on adults. Typical CT signs of PTB in adults include centrilobular nodules, cavities, lymph nodes with calcification, and caseous necrosis (18). Li et al. (19) used cavity features on chest CT to predict multidrug-resistant TB in patients with cavitary disease. Another study showed that a cavity-based radiomics model could predict sputum culture status in MDR-TB patients receiving longer regimens, which could help guide follow-up (20). Also, cavity or consolidation features have been used to tell non-tuberculous mycobacterium lung disease apart from PTB (21, 22). Nodule features have also been used to distinguish lung adenocarcinoma from TB (23).

However, pulmonary diseases in children differ considerably from those in adults, and imaging studies in children are more limited. Few radiomics studies have focused on pediatric lung conditions. One study constructed a nomogram using radiomic features from pulmonary consolidation and mediastinal lymph nodes, along with fever duration, to differentiate PTB from CAP. That nomogram demonstrated excellent performance, with an AUC of 0.977 in the primary cohort and 0.971 in the validation cohort (24). That nomogram worked very well, with AUC 0.977 in the primary cohort and 0.971 in the validation cohort. However, the CAP group included two types of pneumonia, and the proportion of each was not reported, so it’s hard to know if that affected the results. Another study looked at CT radiomics to differentiate Mycoplasma pneumonia (MPP) from SPP in children under 5 years who had similar consolidation patterns (11). The random forest classifier performed best, with an AUC for MPP of 0.822 in the validation cohort.

In our work, we focused on lung consolidation features from CT images. Consolidation is a common finding in children with PTB and CAP, and it is often difficult to distinguish between the two based on visual inspection alone. We attempted to extract more detailed information from consolidation using radiomics. After variance threshold, *t*-tests, and non-parametric tests, 210 features remained. LASSO regression then selected six key features: one first-order feature, four GLCM features, and one GLSZM feature.

The six selected radiomic features may reflect underlying pathological differences. The first-order feature “Mean” captures average CT attenuation within the consolidation; PTB consolidations tend to have lower density due to caseous necrosis, whereas SPP consolidations are dominated by neutrophilic exudates with relatively higher density. The GLCM features (Cluster Shade, Cluster Prominence, Contrast, Difference Variance) describe textural heterogeneity and local intensity variations. Higher contrast in SPP may correspond to more uniform inflammatory exudates, while the mixed areas of necrosis, fibrosis, and granulomatous inflammation in PTB produce more complex texture patterns. The GLSZM feature “Gray Level Non-Uniformity” reflects the homogeneity of gray-level zones; lower uniformity in PTB likely reflects the patchy, non-homogeneous nature of tuberculous consolidation. These texture features capture microscopic tissue heterogeneity not visually appreciable on standard CT.

Among the 10 clinical variables examined, maximum temperature, illness duration, and cough were identified as predictive factors for PTB. Children with PTB had a lower maximum temperature (median 38.5 °C vs. 39.3 °C) and a longer illness duration (median 10 days vs. 5.5 days) compared to those with SPP, consistent with the subacute or chronic course of tuberculosis. Cough was more frequent in the PTB group (28/36 vs. 28/56), which may reflect airway irritation caused by endobronchial tuberculosis or lymph node compression—common features in pediatric PTB that are less prominent in SPP.

We combined the clinical predictors with radiomic signatures using six different machine learning methods to find the best classifier. XGBoost achieved the best performance. The combined radiomic-clinical model performed well in the training cohort (AUC 0.981, 95% CI 0.878–0.982) and the validation cohort (AUC 0.897, 95% CI 0.672–0.927).

There are some limitations here. First, it’s a single-center study with a small sample size. More prospective, multi-center studies are needed to confirm our findings. Second, we only extracted radiomic features from lung consolidation and did not include lymph nodes from CT images. Future studies could try a more comprehensive approach. Third, this study is retrospective in design, and selection bias may exist among children who underwent CT examination. In our clinical practice, CT is not routinely performed for all children with suspected pneumonia or tuberculosis; it is primarily reserved for specific situations, including those who do not respond to empirical anti-infective therapy, those with suspected pulmonary tuberculosis but negative microbiological findings, or those with a definite history of tuberculosis exposure. Therefore, the target population of our model should be limited to this diagnostically challenging subgroup rather than all children with respiratory infections. Whether the model can be applied for prospective prediction requires further multicenter and prospective validation. Importantly, this model is not intended to replace microbiological testing, but may serve as an adjunctive decision-support tool when etiological results are delayed or negative.

In summary, we developed and internally validated a CT-based model that combines clinical characteristics and radiomic features to distinguish PTB from SPP. The XGBoost-based radiomic-clinical model showed the best diagnostic performance, and could offer a fast, non-invasive tool for clinical decision-making.

## Data Availability

The original contributions presented in the study are included in the article/supplementary material, further inquiries can be directed to the corresponding authors.

## Footnotes

1
http://www.Slicer.org
